# Immunomodulatory effect of efferocytosis at the maternal–fetal interface

**DOI:** 10.1186/s12964-025-02055-9

**Published:** 2025-01-27

**Authors:** Hui Tao, Ruilin Ma, Jianjian Cui, Zejun Yang, Wencong He, Yanan Li, Yin Zhao

**Affiliations:** 1 Department of Obstetrics and Gynecology, Union Hospital, Tongji Medical College, Huazhong University of Science and Technology, No. 1277 Jiefang Avenue, Wuhan, 430022 China; 2 Shenzhen Huazhong University of Science and Technology Research Institute, Shenzhen, 518000 China; 3 Department of Prenatal Diagnosis Center, Union Hospital, Tongji Medical College, Huazhong University of Science and Technology, No. 1277 Jiefang Avenue, Wuhan, 430022 China

**Keywords:** Efferocytosis, Immune, Metabolism, Inflammation, Preeclampsia, Recurrent spontaneous abortion

## Abstract

Efferocytosis is a mechanism by which phagocytes efficiently clear apoptotic cells, averting their secondary necrosis and the subsequent release of potentially immunogenic or cytotoxic substances that can trigger strong immune and inflammatory responses. During efferocytosis, the metabolic pathways of phagocytes are transformed, which, along with the catabolism of apoptotic cargo, can affect their function and inflammatory state. Extensive apoptosis occurs during placental development, and some studies reported the immunomodulatory effects of efferocytosis at the maternal–fetal interface. The dysregulation of efferocytosis is strongly linked to pregnancy complications such as preeclampsia and recurrent spontaneous abortion. In this review, we discuss the mechanisms of efferocytosis and its relationships with metabolism and inflammation. We also highlight the roles of professional and non-professional phagocytes in efferocytosis at the maternal–fetal interface and their impact on pregnancy outcomes and explore relevant regulatory factors. These insights are expected to guide future basic research and clinical strategies for identifying efferocytosis-related molecules as potential predictors or therapeutic targets in obstetric diseases.

## Introduction

Efferocytosis refers to the timely and effective clearance of apoptotic cells (ACs) by phagocytes. It prevents the secondary necrosis of ACs, averting the leakage of potential immunogenic or cytotoxic substances and mitigating severe immune and inflammatory responses [1]. During placental development, extensive apoptosis exists, particularly among trophoblast, which benefits trophoblast differentiation, immune tolerance and spiral artery remodeling [2]. However, apoptosis must be well controlled in case that excessive apoptosis results in cell function loss and secondary necrosis. The regulatory mechanisms of apoptosis encompass: the production and elimination of ACs, with the latter involving efferocytosis. Professional phagocytes, including macrophages and dendritic cells, are key players in AC clearance. Non-professional phagocytes, such as epithelial cells, fibroblasts, endothelial cells, and mesenchymal cells, are distributed throughout various organs and generally undergo efferocytosis in situ. In scenarios in which macrophages are absent or their recruitment is delayed, non-professional phagocytes proximal to the lesion site assume the role of primary phagocytes [3, 4].

**Fig. 1:**
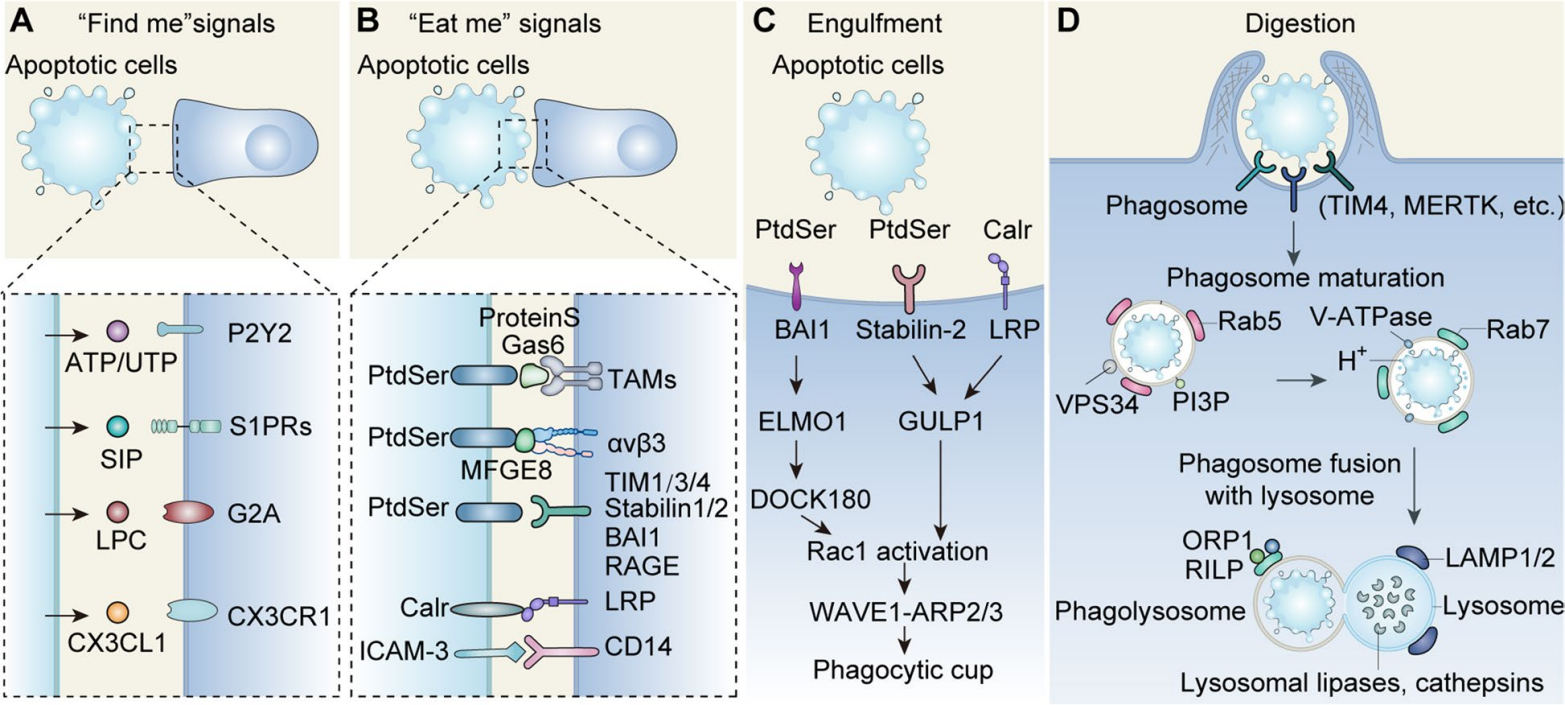
The steps of efferocytosis. Effective efferocytosis involves four steps: “find me”, “eat me”, engulfment and digestion. **A** Apoptotic cells release “find me” signals that target receptors on phagocytes, stimulating their migration to the site of cell death. **B** “Eat me” signals, as cell surface ligands on apoptotic cells, directly or via bridge molecules, interact with engulfment receptors on phagocytes to facilitate the subsequent engulfment process. **C** Phagocytes internalize cell corpses via cytoskeletal rearrangement, which involves Rac1 activation trigged by the DOCK180/ELMO1 set. **D** The digestion of apoptotic cargo in phagocytes is conducted by phagosome maturation and fusion with lysosome, and leads to the secretion of anti-inflammatory cytokines

Effective efferocytosis consists of four steps: “find me”, “eat me”, “engulfment” and “digestion” [5] (Fig. 1). ACs emit “find me” signals that target receptors on phagocytes, encouraging them to move toward the site of apoptosis. The classic “find me” signals include nucleotides (ATP/UTP/AMP) [6], sphingosine-1-phosphate (S1P) [7], lysophosphatidylcholine (LPC) [8, 9] and C-X-C motif chemokine ligand 1 (CX3CL1) [10]. In the second step of efferocytosis, “eat me” signals, as cell surface ligands on ACs, directly or via bridge molecules (e.g., growth arrest-specific gene 6 (GAS6), protein S, and milk fat globule epidermal growth factor 8 (MFGE8)), interact with engulfment receptors on phagocytes to facilitate the engulfment process. Phosphatidylserine (PtdSer) is the most extensively researched “eat me” signal, which can be identified by multiple “eat me” receptors, including the CD300 family, stabilin 1/2, brain-specific angiogenesis inhibitor 1 (BAI1), T cell immunoglobulin mucin receptor (TIM) 1/3/4, receptor for advanced glycation end products (RAGE) [11–13], TYRO3, AXL and MerTK receptors [14], integrin ανβ3 receptors [15]. “Don’t eat me” signals (e.g., CD31, CD47, and CD24) are surficial ligands expressed on living cells that help them escape efferocytosis; these signals are diminished in ACs [16]. After recognizing and binding to ACs, phagocytes initiate actin aggregation and rearrangement, causing the plasma membrane to invaginate and form phagosomes [17]. The RHO family of small GTPases, particularly RAC1, mediate this engulfment process. During digestion, phagosomes mature and fuse with lysosomes to degrade cell corpses using various lipases, nucleases, and proteases in lysosomes [18]. These decomposition products derived from ACs can reprogram macrophage metabolism with functional consequences for the cell.

## Immunomodulatory effect of efferocytosis

### Anti-inflammatory response and metabolic reprogramming in macrophages during efferocytosis

#### Anti-inflammatory response after macrophage efferocytosis

The maintenance of tissue homeostasis via efferocytosis depends on its immunomodulatory effect. Efferocytosis is generally believed to play an anti-inflammatory and pro-resolving role, particularly in macrophage-mediated phagocytosis, for two reasons. First, efferocytosis enables the engulfment of ACs while their plasma membranes remain intact. This prevents secondary necroptosis, which can induce inflammation due to the leakage of cytotoxic and immunogenic intracellular contents such as damage-associated molecular patterns. Second, efferocytosis directly modifies inflammatory signaling pathways in engulfing phagocytes [19]. For example, at the “find me” stage, ACs release ATP and UTP to recruit macrophages; these extracellular nucleotides also exert an immunomodulatory influence on macrophages. ATP or AMP can be broken down into adenosine, which inhibits pro-inflammatory cytokines such as CXCL1 and CXCL2 and upregulates pro-resolving factors such as Nr4a1, Nr4a2 and thrombospondin via adenosine receptors on macrophages [20]. Different stages of efferocytosis are regulated by various signaling molecules, some of which mediate the immunomodulatory effects of ACs on macrophages (Table 1).

**Table 1:** Immunomodulatory roles of efferocytosis signals in macrophages

Efferocytosis stages	Molecules	Immunomodulatory roles	Ref
“Find me” signals	ATP/UTP/AMP	• Catabolite adenosine inhibits pro-inflammatory cytokines (e.g. CXCL1, CXCL2) and upregulates pro-resolution factors (e.g. Nr4a1, Thbs1) via adenosine receptors Gs-linked A2a and A2b	[21]
	CX3CL1/CX3CR1	• Reduce expression of inflammatory cytokines and enhance expression of pro-survival (e.g. BCL-2) and antioxidant genes (HO-1)	[22–24]
	S1P/S1PR	• S1P suppresses the production of TNF and IL-12 and increases the production of IL-10, VEGF and PGE2	[25–27]
		• S1P can activate nuclear receptor PPARγ	[28]
		• S1PR promotes M2 phenotype, including increased cAMP and PTGS2 and suppression of NFkB signaling	[29–31]
	LPC/G2A	• Reduce IL-6 level	[32]
“Eat me” signals	BAI1	• BAI1 deletion augments IL-1α, IL-6 and TNF	[33]
	TIM (TIM-1, 3, 4)	• TIM1 and TIM3 inhibit NFkB activation and inflammatory cytokine production (e.g. TNF, IL-6, CCL5)	[34, 35]
		• TIM-4 can trigger LC3-associated phagocytosis (LAP)	[36]
		• TIM-4 regulates Toll-like receptor response by upregulating IL-10 and downregulating TNF-α productions	[37]
	CD300 (CD300A, CD300B, CD300F)	• Can trigger either activating or inhibitory immunomodulatory outcomes depending on their association with different signaling modules	[19]
	Stabilin receptors (Stab1, Stab2)	• Stabilin2 stimulates TGFβ production	[38]
	MFGE8/ανβ3 or ανβ5	• MFEG8 increases levels of pro-inflammatory TNF-α and decreases levels of anti-inflammatory IL-10	[39]
		• αν–mediated apoptotic cell recognition has significant effects on macrophage TNF and IL-10 production	[40, 41]
	TAMs (Tyro3, Axl, Mer)	• Signaling via Axl and Mer can directly suppress TLR- and type I IFN-driven inflammatory signaling pathways (e.g. TLR4-NFκB-TNF, TLR4-Twist-TNF, IFNα- STAT1-SCOS1/3)	[42–45]
	Gas6/Protein S	• rGas6 reduces the secretion of TNF-α, IL-1β, and macrophage inflammatory protein-2, and increases the secretion of hepatocyte growth factor	[46, 47]
		• Pros1 induces higher levels of IL-10, and lower levels of TNFα and CCL3	[48]
	LRP-1	• Silencing LRP-1 reduces efferocytosis and restores inflammatory gene expression (MCP-1, IL-1β, and IL-12) in PKM2-deficient macrophages	[49]
	CD14	• Cross-linked CD14 triggers MerTK phosphorylation and MerTK-mediated IL-10 secretion	[50]
Engulfment	BELMO (fusing BAI1 and C-term of ELMO1)	• BELMO increases ER-resident enzymes and chaperones to overcome protein-folding-associated toxicity, and produces less pro-inflammatory cytokine (TNF, IL-16, MCP-1) and more IL-10 to control inflammation	[51]
Digestion	the nuclear receptor (NR) family (LXR, PPAR)	• Induce TGFβ and IL-10 production and suppress IL-1β, TNF and IL-12 production	[52, 53]
	ABCA1	• Decrease TNFα and IL-6 levels by binding to apoA-I	[54]

**Fig. 2:**
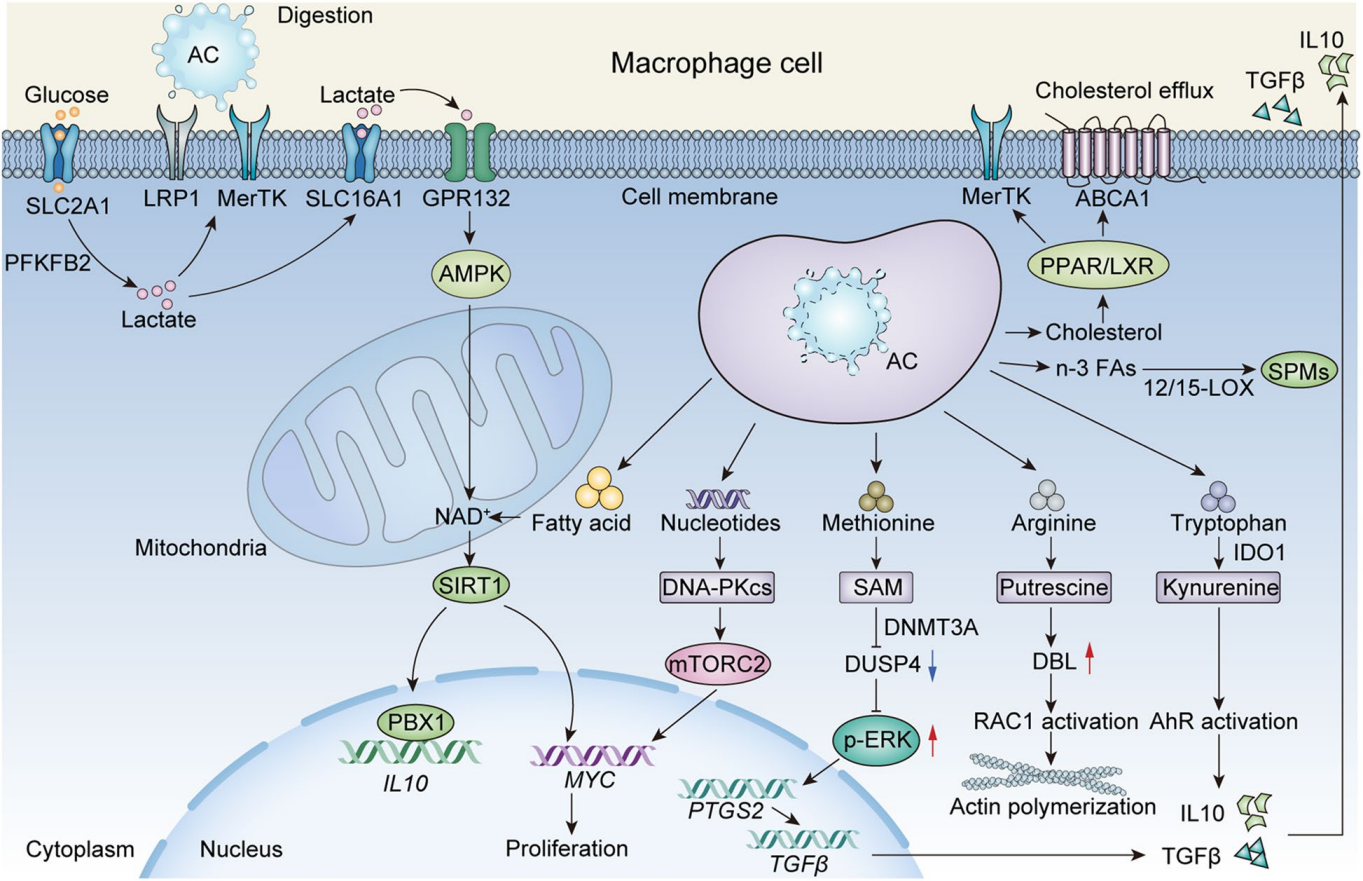
The metabolic dynamics of efferocytosis. During the digestion stage of efferocytosis, metabolites derived from apoptotic cells modulate metabolic signaling in macrophages to promote inflammation resolution and continuous efferocytosis. Cholesterol is excreted via ABCA1 to restore macrophage metabolic homeostasis. The upregulation of ABCA1 is dependent on nuclear receptors LXR and PPARγ, which inhibit the transcription of inflammatory genes and induce MerTK expression. Efferocytosis upregulates the expression of 12/15-LOX, leading to increased generation of long-chain fatty acid-derived lipids called SPMs. The mitochondrial oxidation of fatty acids produces NAD + , which triggers SIRT1 deacetylase, inducing the binding of PBX1 to the IL10 gene promoter and promoting its expression. Arginine is converted into putrescine, which aids in RAC1 activation, driving subsequent rounds of efferocytosis. SAM, a metabolite from methionine, represses DUSP4 expression and restores ERK phosphorylation, activating the PTGS2-PGE2-TGFβ1 pathway. Kynurenine, produced from tryptophan by IDO1, increases the expression of IL10 and TGFβ and augments continual efferocytosis by activating AhR. Apoptotic cell DNA is hydrolyzed into nucleotides, which enhances MYC activity through the DNA–PKcs–mTORC2/Rictor pathway. This process stimulates the proliferation of non-inflammatory macrophages, thereby benefiting the resolution of injury. SLC2A1 enhances glucose uptake and glycolysis, promoting ATP generation and actin polymerization, which ensures continuous efferocytosis. SLC16A1-mediated lactate release drives the generation of anti-inflammatory factors such as IL10. Lactate also promotes efferocytosis-induced macrophage proliferation by activating MYC and facilitates continual efferocytosis by upregulating MerTK and LRP1

Notably, during the “digestion” stage, the metabolite load from ACs can alter the metabolic pathways and downstream function of macrophages. Some of these metabolites (e.g., cholesterol [55, 56]) are excreted to restore macrophage metabolic homeostasis, while others (e.g., amino acids [57–59], fatty acids and nucleosides [60]) are further processed by macrophages, inducing continual efferocytosis and resolving inflammation (Fig. 2). For example, the oxidation of AC-derived fatty acids produces NAD + , which activates Sirtuin1 deacetylase. This activation triggers pre-B cell leukemia transcription factor (PBX)1 binding to the interleukin (IL)10 promoter, thereby promoting IL10 expression [61].

#### Immunometabolism of macrophages during efferocytosis

Efferocytosis is a highly energy-demanding process that requires phagocytes to adjust intracellular metabolic pathways and ensure a sufficient energy supply for finding, engulfing, and digesting ACs. The transformation of metabolic pathways in phagocytes can affect their function and inflammatory state. Here, we discuss the transition between oxidative phosphorylation (OXPHOS) and glycolysis and their synergistic effect in phagocytes during efferocytosis.

A recent study revealed the interdependent relationship between glycolysis and efferocytosis. The solute carrier (SLC) family, a class of transmembrane proteins, is responsible for metabolite transport. Some SLC members are modified and utilized during efferocytosis. At the “find me” stage, secretions from ACs induce the expression of the enzyme SGK1 in phagocytes, facilitating the transport of SLC2A1 (a glucose transporter) to the plasma membrane. At the “eat me” stage, the binding of PtdSer to its receptors upregulates SLC2A1 expression in phagocytes. SLC2A1 enhances glucose uptake and glycolysis, promoting ATP generation and actin polymerization, thereby ensuring continuous efferocytosis, in vitro and in vivo. At the “digestion” stage, the internalization of ACs increases lactate transporter SLC16A1 level. SLC16A1-mediated lactate release drives the production of anti-inflammatory factors such as IL10 [62]. Lactate also promotes efferocytosis-induced macrophage proliferation to achieve continuous efferocytosis. This process is mediated by the lactate receptor GPR132, which supports MYC protein stability via an AMPK-NAD + -SIRT1 pathway [63]. Coincidentally, 6-phosphofructo-2-kinase/fructose-2,6-bisphosphatase 2 (PFKFB2, a glycolysis rate-limiting enzyme) reportedly participates in efferocytosis-induced macrophage glycolysis and boosts lactate-driven continual efferocytosis by upregulating the expressions of efferocytosis receptors MerTK and LRP1 [64]. Growing evidence has demonstrated that glycolysis fuels enhanced efferocytosis [65, 66]. Nevertheless, Doddapattar et al. found that silencing the glycolytic enzyme pyruvate kinase muscle 2 (PKM2) in myeloid cells or restricting its nuclear translocation inhibited glycolysis but promoted efferocytosis and relieved atherosclerosis [49].

Mitochondrial dynamics within phagocytes also contribute to constant efferocytosis and the maintenance of cellular homeostasis. For instance, the mitochondrial membrane potential (MMP) increases upon AC internalization. Subsequently, the expression of the mitochondrial uncoupling protein, UCP2, is upregulated to protect against excessive MMP. In vitro and in vivo experiments verified that the deletion of UCP2 maintains MMP at a high level and impairs initial and continuous efferocytosis [67]. Similarly, a compromised macrophage phagocytic capacity was observed in mice after knockout of the gene encoding the mitochondrial fission protein dynamin-related protein 1 (DRP1). DRP1-mediated mitochondrial fission causes the release of endoplasmic reticulum calcium into the cytoplasm rather than the mitochondria, prompting vesicular transport of the phagolysosome to the plasma membrane and allowing phagosome formation around subsequent ACs [68]. Furthermore, Cai et al. reported that myeloid-specific deletion of the mitochondrial complex I protein encoded by Ndufs4 shifts macrophages toward a pro-inflammatory metabolic phenotype and exaggerates their response to lipopolysaccharide. The mechanism involves deficient efferocytosis and reduced expression of anti-inflammatory factors [69].

Taken together, these findings suggest that both glycolysis and OXPHOS support efferocytosis. However, it remains uncertain which pathway plays the dominant role. Glycolysis was previously thought to promote inflammation [70]; however, recent studies demonstrated its pro-resolving effects [71, 72]. This seemingly contradictory evidence indicates that glycolysis may be required for both pro-inflammatory and pro-resolving processes in macrophages [73]. Resolution is the body’s instinctive response to inflammation; therefore, inflammation-induced glycolysis may serve as an initiating factor for the resolution cascade [74]. Alternatively, glycolysis is required by phagocytes to manage the increased metabolite load caused by AC engulfment. Morioka et al. identified a genetic program that is active during efferocytosis marked by an increased expression of genes associated with glycolysis and a reduced expression of genes related to OXPHOS. This study also highlighted the role of lactate, the final product of glycolysis, in promoting an anti-inflammatory circumstance [62]. Notably, this study focused on the link between glycolysis and efferocytosis during only the initial few hours. Given that mitochondrial respiration is the main metabolic pattern for pro-resolving macrophages [70], the role of OXPHOS in the later stages of efferocytosis cannot be ruled out. Thorp et al. showed that both glycolysis and OXPHOS are enhanced during the process of efferocytosis; however, macrophages rely more on the latter to produce pro-resolving cytokines [61]. These findings suggest that different steps of efferocytosis may depend on distinct metabolic patterns [75]. The intricate regulation of metabolism during efferocytosis and its effect on tissue homeostasis warrant further research.

### Inflammatory/anti-inflammatory response after non-professional phagocyte efferocytosis

Efferocytosis mediated by non-professional phagocytes can exert inflammatory or anti-inflammatory effects depending on the tissue microenvironment. For instance, kidney epithelial cells can phagocytose apoptotic and necrotic cells following kidney injury, exhibiting an anti-inflammatory phenotype by downregulating nuclear factor-kappa B (NF-κB) activity. This leads to decreased pro-inflammatory cytokine secretions and impaired macrophage activation, collectively promoting kidney repair [34, 76]. Furthermore, the defective efferocytosis of aortic epithelial cells reportedly downregulates endothelial nitric oxide synthase (eNOS) expression and upregulates NF-κB, mitogen-activated protein kinase (MAPK) subunits, and pro-inflammatory cytokine expressions, resulting in vascular dysfunction and accelerated vascular aging [77]. Conversely, a recent study revealed that the phagocytosis of apoptotic endothelial cells by neighboring microvascular endothelial cells triggers the synthesis of pro-inflammatory cytokines and promotes leukocyte adhesion [78]. Similarly, the engulfment of myelin debris by endothelial cells stimulates inflammation, pathological angiogenesis and fibrosis, contributing to the progression of demyelination disorders after neural injury [79]. On the one hand, these conflicting views (anti- vs. pro-inflammatory) are due to differences in the tissue microenvironment at the site of efferocytosis; on the other hand, they are also due to differences in the AC origin or activation mechanism [80]. For example, human umbilical vein endothelial cells can be induced to undergo apoptosis and develop into two types under different stimuli. One type is the apoptotic body (1–3 μm, AnnexinV + /DAPI + /histone +) that contains IL1α and is capable of inducing the chemokines IL8 and monocyte chemoattractant protein 1 (MCP1). The other type is the microparticle (< 1 μm, AnnexinV + /DAPI-/histone-) that lacks IL1α and is thus unable to induce pro-inflammatory chemokines [81].

## Professional and non-professional phagocytes at the maternal–fetal interface

### Efferocytosis and macrophages

Macrophages, which are present in nearly all tissues, perform critical functions including recognizing, ingesting and processing of foreign materials, dead cells, and other debris. Macrophages originate from monocytes and are recruited to inflammatory sites for immune defense and homeostasis maintenance [82]. Under various stimuli, macrophages exhibit two main phenotypes: classically activated M1 and alternatively activated M2. M1 macrophages are pro-inflammatory and play a crucial role in the early stages of inflammation by recruiting Th1 cells and engulfing pathogens. In contrast, M2 macrophages are pro-resolving and function primarily during the later stages of inflammation by clearing debris, inhibiting inflammation, and promoting angiogenesis and tissue repair [83]. Although increasing evidence suggests that tissue-resident macrophages may not strictly conform to this classification owing to environmental variability, this dichotomy remains a useful framework for understanding macrophage functions in the context of diseases [84].

Decidual macrophages (dMφ), derived from maternal monocytes, are recruited to the decidua from the peripheral blood. These cells play critical roles in embryo implantation, placental vascular remodeling, and immune regulation at the maternal–fetal interface. During early pregnancy, dMφ are the second largest leukocyte population surpassed only by uterine nature killer (uNK) cells, and their numbers remain high until the third trimester. dMφ predominantly exhibit the M1 phenotype in the first trimester, supporting implantation and placentation. In the second trimester, they transition to the M2 phenotype to promote tolerance toward the semi-allogeneic fetus. In the third trimester, dMφ revert to the M1 phenotype, preparing for the initiation of labor [85].

dMφ are the main phagocytes in charge of eliminating dead cells. During the trophoblast-independent stage of spiral artery remodeling, vascular smooth muscle cells (VSMCs) are disrupted following the infiltration of uNK cells and dMφ [86]. Subsequently, apoptotic VSMCs are phagocytosed by dMφ to prevent their secondary necrosis and the release of pro-inflammatory substances. This process is driven by upregulation of the “find me” signal fractalkine, externalization of the “eat me” signal calreticulin on apoptotic VSMCs, and increased expression of the “eat me” signal receptor LRP1 on macrophages [87]. When extravillous trophoblasts (EVTs) invade the decidua, most decidual stromal cells (DSCs) and trophoblasts undergo apoptosis. Both trophoblasts and trophoblast debris are sources of fetal minor histocompatibility antigens that can trigger the rejection of human leukocyte antigen (HLA)-matched organ grafts [88]. Hence, it is essential to promptly and effectively remove these potential risks to the human fetal allograft.

Macrophages and endothelial cells are responsible for clearing the apoptotic trophoblasts and trophoblast debris [89]. Macrophages tend to adopt a tolerant phenotype after phagocytosis characterized by the decreased expressions of MHC-II molecules, costimulatory molecules (CD80, CD86, CD40 and B7H3), MCP1, intercellular adhesion molecule 1 (ICAM1), and IL8 receptors along with increased expression of programmed death-1 ligand 1 (PD-L1). Furthermore, the phagocytosis of trophoblast debris promotes release of the anti-inflammatory cytokines IL10 and IL1Ra while inhibiting release of the pro-inflammatory cytokines IL1β, IL12p70 and IL8 by macrophages. This may contribute to maternal tolerance of the fetal allograft, enabling its survival [90].

Nevertheless, in pregnancies complicated by preeclampsia, more trophoblasts die, most of which are necrotic, and phagocytosis can induce an inflammatory response [91]. Mercnik et al. found that placental macrophages in the setting of early-onset preeclampsia switch toward M1 polarization but exhibit high phagocytic activity and inflammation-resolving capacity [92]. These conflicting perspectives may be attributed to the different stages of preeclampsia. Numbers of apoptotic trophoblasts increase dramatically in preeclampsia placentas, and efferocytosis by macrophages is concomitantly enhanced to alleviate inflammation. However, this process cannot clear all of the apoptotic trophoblasts, leading to further necrosis and inflammation. Another recent study showed that macrophage engulfment of DSCs is modulated by the IL33/ST2 axis, which not only affects macrophage polarization and AXL-mediated efferocytosis, but also regulates macrophage metabolic reprogramming. Disruption of the IL33/ST2 axis leads to immune metabolic disorders and pregnancy loss [66].

Research is limited regarding the role of dMφ during late pregnancy. The initiation of labor requires an inflammatory environment; however, the inflammatory response should not be too strong to prevent preterm delivery. dMφ play a significant role in promoting cervical ripening and balancing pro- and anti-inflammatory factors [85]. Furthermore, dMφ are involved in postpartum uterine remodeling and tissue homeostasis maintenance via AC clearance and wound healing accompanied by the secretion of anti-inflammatory cytokines. dMφ isolated from term placentas have been discovered to produce and secret transforming growth factor (TGF) β and display a high capacity for phagocytosing ACs, a process facilitated by vasoactive intestinal peptide (VIP) [93]. Taken together, efferocytosis by dMφ spans the entire pregnancy process, mediating maternal immune tolerance and tissue repair.

### Efferocytosis and endothelial cells

During uterine artery remodeling, EVTs invade the decidual and inner myometrium and come into close contact with human endometrial microvascular endothelial cells (HEECs). HEECs are proven to phagocytose apoptotic trophoblasts, a process that can be suppressed by the phagocytosis inhibitor cytochalasin B. Peng et al. found that efferocytosis of apoptotic trophoblasts augments the release of pro-inflammatory cytokines IL6 and MCP1 by HEECs, which may contribute to the mild inflammatory state at the maternal–fetal interface during early pregnancy. Furthermore, they deduced that, in parallel with the exaggeration of trophoblast apoptosis in preeclampsia, the phagocytosis of apoptotic trophoblasts by HEECs is enhanced, resulting in an amplified inflammatory response, which might be one of the pathogenic mechanisms of preeclampsia [94].

At the end of trophoblast life cycle, its debris is shed from the placental surface into the maternal blood, expanding the maternal–fetal interface from the placenta to the whole body. Trophoblast debris flows through the blood circulation and is ultimately trapped within smaller pulmonary capillaries. As mentioned above, macrophages are the most promising candidates for the phagocytosis of trophoblast debris; however, pulmonary macrophages reside in the airways instead of the blood vessels and have no access to trophoblast debris. Consequently, the endothelial cells of the maternal pulmonary vessels become the primary phagocytes to engulf the trophoblast debris. Trophoblast debris from normal pregnancy exhibits apoptotic properties, whereas that from preeclampsia shows more necrotic characteristics [91]. The phagocytosis of necrotic trophoblasts promotes endothelial cell activation, characterized by elevated ICAM1 expression and enhanced monocyte adhesion [95]. Meanwhile, these endothelial cells secrete more IL6 and TGFβ1, which can spread cellular activation from the initial phagocytic site to distant endothelial cells [96, 97]. In other words, the phagocytosis of necrotic trophoblast debris within the pulmonary capillaries triggers systemic activation of maternal endothelium, a hallmark of preeclampsia, via the secretion of soluble factors such as IL6. Considering that the oxidative burst during phagocytosis might be a reason for phagocyte activation, Chen Q et al. demonstrated that the antioxidant vitamin C inhibits the activation of endothelial cells that have ingested necrotic trophoblasts and hinders the increased IL6 secretion [98]. Calcium channel blockers have a similar effect to vitamin C; thus, nifedipine has become a second-line treatment for preeclampsia [99]. However, the phagocytosis of apoptotic trophoblastic debris does not activate endothelial cells; rather, it protects them from activation by necrotic trophoblast debris, IL6, lipopolysaccharide, or phorbol myristate acetate [100]. This protective mechanism enables maternal vessels to adapt to normal physiological changes that occur during pregnancy.

### Efferocytosis and trophoblasts

Trophoblasts, epithelial cells of fetal origin, display potent phagocytic activity during the peri-implantation period [101]. This activity was implicated in the nutritive significance of trophoblasts, which was determined many years ago. Most notably, trophoblasts phagocytose erythrocytes to extract protein and iron for fetal hemopoiesis. Progesterone and VIP were recently shown to intensify erythrocyte phagocytosis by trophoblasts and induce an anti-inflammatory profile [102]. During the process of spiral artery remodeling, trophoblasts invade the decidua and establish contact with uterine epithelial and decidual cells, which are regarded as the maternal barrier along the invasion pathway. Invading trophoblasts induce the apoptosis of epithelial and decidual cells and phagocytose them, ensuring successful completion of vascular transformation [103]. Trophoblast phagocytosis is also involved in immune defense mechanisms. Trophoblast cells can engulf microorganisms present at the maternal–fetal interface to control the spread of infection and inflammation in the mother and fetus. For instance, inadequate trophoblast phagocytosis has been identified as a significant factor in the pathogenesis of chorioamnionitis [104].

## Efferocytosis and complications of pregnancy

**Fig. 3:**
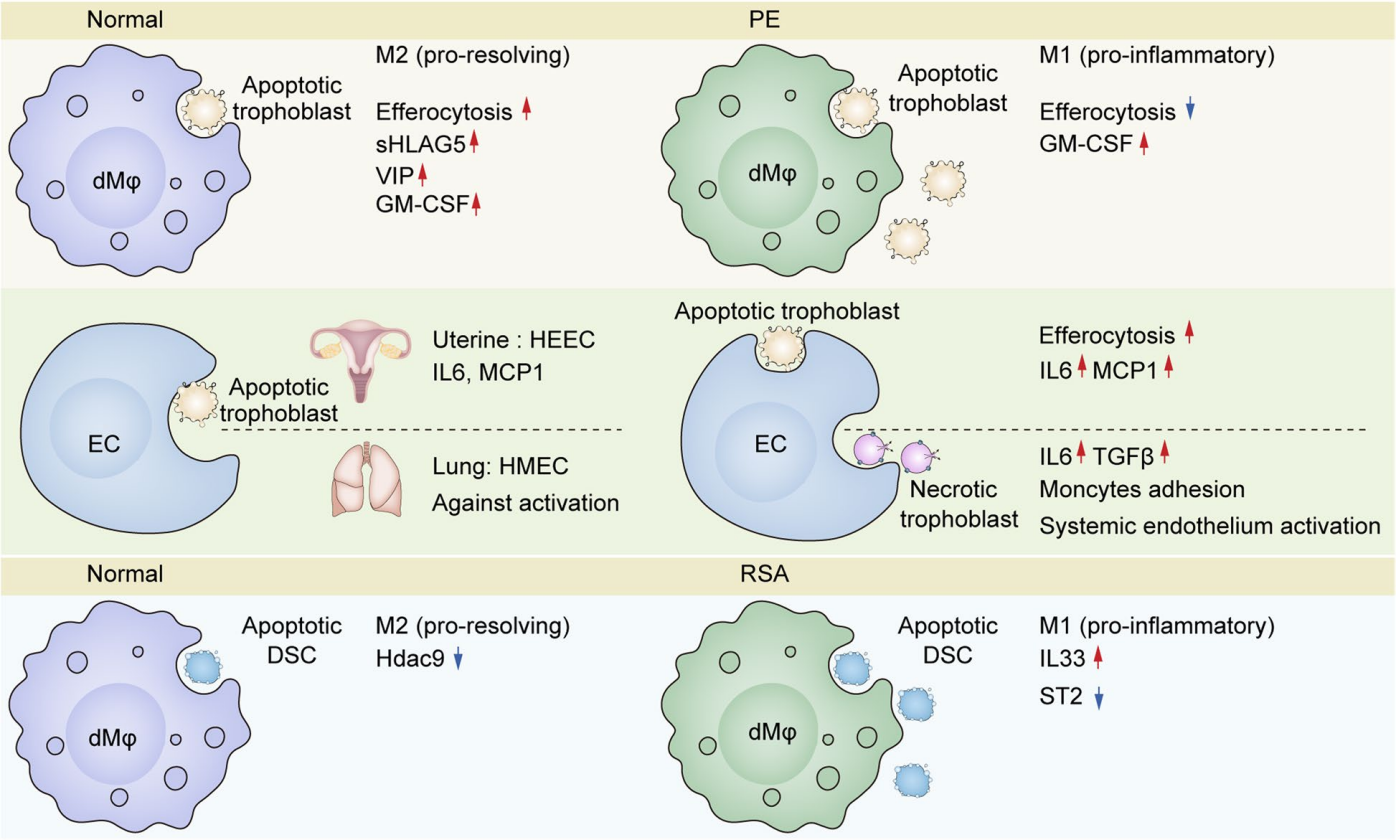
Efferocytosis in pregnancy complications and relevant regulatory factors. Preeclampsia (PE) and recurrent spontaneous abortion (RSA) are typical pregnancy complications associated with dysfunctional efferocytosis. In PE, the apoptosis of trophoblasts increases, and their clearance is mainly carried out by macrophages and endothelial cells. Macrophages, as professional phagocytes, can be categorized into M1 and M2 phenotypes: M1 macrophages primarily defend against pathogens, while M2 macrophages are involved in inhibiting inflammation, clearing debris, and promoting angiogenesis. In PE, macrophages transform into the M1 type, accompanied by decreased phagocytic capacity and increased release of pro-inflammatory factors. sHLAG5 and VIP, secreted by trophoblast cells, and M-CSF, secreted by decidual cells, promote M2 polarization of macrophages and enhance their phagocytic activity. Conversely, GM-CSF secreted by decidual cells polarizes macrophages towards the M1 subtype. Human endometrial microvascular endothelial cells (HEECs), as non-professional phagocytes, assist in engulfing apoptotic trophoblasts and release pro-inflammatory IL6 and MCP1, which may accelerate the occurrence and progression of PE. Trophoblast debris detaching from the placenta into maternal blood circulation is primarily removed by maternal pulmonary vascular endothelial cells. The phagocytosis of apoptotic trophoblasts does not activate pulmonary vascular endothelial cells, whereas the phagocytosis of necrotic trophoblasts does. In PE, trophoblasts mainly exhibit necrotic characteristics, and their phagocytosis induces local and distant endothelial cell activation through the secretion of IL6 and TGFβ. The pathogenesis of RSA is related to an imbalance between M1 and M2 macrophages, in which Hdac9 and IL33/ST2 axis play a regulatory role

Normal pregnancy is a state of immunosuppression and mild inflammation in which efferocytosis, as described above, plays an important role. The dysregulation of efferocytosis can cause excessive activation of immunity and inflammation, leading to pregnancy complications such as preeclampsia [105] and recurrent spontaneous abortion [89] (Fig. 3).

### Efferocytosis and preeclampsia

Preeclampsia is a progressive multisystem disease unique to pregnancy, featured by new-onset hypertension and proteinuria, or hypertension accompanied by multiple organ dysfunctions, such as thrombocytopenia, elevated liver enzymes, renal insufficiency, pulmonary edema, neurological abnormalities or visual impairments, occurring after 20 weeks of gestation [106]. This condition can progress rapidly, posing a serious risk to both maternal and fetal health, with a prevalence rate up to 5–7% [107]. The fundamental pathophysiological changes in preeclampsia include systemic small vessel spasm and vascular endothelial damage. Although the exact cause of preeclampsia remains unclear, excessive activation of inflammation and immunity is known to be a key factor in its pathogenesis. The apoptosis of trophoblasts increases in preeclampsia, and their clearance is mainly performed by macrophages and endothelial cells. Macrophages, as professional phagocytes, can be categorized into M1 and M2 phenotypes, which differ in their cell markers, secreted cytokines and biological functions. Briefly, M1 macrophage is pro-inflammatory, while M2 macrophage is pro-resolving and dominates efferocytosis [108]. In preeclampsia, there is an imbalance between M1 and M2 macrophages, but opinions on which phenotype is dominant are inconsistent. Most scholars believe that in preeclampsia, macrophages transform into the M1 type, accompanied by decreased phagocytic capacity and increased release of pro-inflammatory factors [109–112]. However, Tsai suggests that macrophages in preeclampsia transform into the M2 type, with increased IL10 secretion but weakened phagocytic capacity. The proposed mechanism is that the reduced SIK3 expression in trophoblast cells leads to increased CCL24 release, which in turn promotes a skewed M2 macrophage phenotype in the decidua. M2 macrophages secrete a large amount of IL10, inhibiting trophoblast invasion and migration. Meanwhile, IL10 stimulates trophoblasts to generate more CCL24, maintaining the dominance of M2 macrophages in preeclampsia placental environment. As for the contradictory viewpoint that M2 macrophages dominate but have a weakened phagocytic capacity in preeclampsia, this article does not provide an explanation [113]. Mercnik further subdivided preeclampsia into early-onset and late-onset types, comparing the polarization characteristics and functional changes of placental macrophages between them and normal pregnancy. Macrophages in both early- and late-onset preeclampsia exhibit M1 and M2 characteristics, but the shift toward the M1 phenotype is more prominent in the former. M1 biomarkers, including CD86, TLR4, and HLA-DR, are significantly upregulated in early-onset preeclampsia, and the high expressions of IRF5 and NOS2 also confirm this pro-inflammatory profile. In contrast, M2 features are more pronounced in late-onset preeclampsia, although the levels of pro-inflammatory markers CD80 and TLR1 are raised in comparison with normal pregnancy. The enhanced phagocytic capacity of placental macrophages in early- and late-onset preeclampsia, along with their elevated secretions of MMP9 and anti-inflammatory cytokines IL4, IL13, and TGFβ, indicate their adaptive role and plasticity in managing inflammation and maintaining tissue homeostasis [114], which can be understood as a compensatory effect on preeclampsia.

More studies have explored the factors influencing the phenotypic transition of dMφ. For example, soluble human leukocyte antigen G5 (sHLAG5) secreted by trophoblast cells promotes M2 polarization of macrophages and enhances their phagocytic activity. Polarized macrophages, in turn, suppress T cell proliferation and interferon γ (IFNγ) production in T cells, and promote trophoblast invasion, thus regulating maternal–fetal tolerance and placental development [115]. Trophoblast cells can also secrete VIP (as mentioned in “Efferocytosis and macrophages” section) [93], which promotes the phagocytosis of apoptotic trophoblasts by monocytes and increases the expression of anti-inflammatory factors IL10 and CD39 in monocytes. This phenomenon is attributed to the enhanced expression and re-orientation of integrin ανβ3 on phagocytes and increased expression of thrombospondin 1 on trophoblasts under VIP stimulation. Integrin ανβ3 and thrombospondin 1 play crucial roles in creating a phagocytic channel for the immune-silenced clearance of ACs [116]. Moreover, monocytes of pregnant women exhibit increased glycolysis and enhanced efferocytosis compared to those from non-pregnant controls. In vitro treatment of monocytes from non-pregnant women with conditioned medium from human first-trimester trophoblasts induced an immunometabolic phenotype similar to that of pregnant monocytes. This finding suggests that trophoblasts may promote the efferocytotic capacity of monocytes by altering their metabolic phenotype [117]. The chemokines secreted by decidual cells also significantly impact macrophage differentiation. Li et al. found that: 1) the preeclampsia decidua contains excessive macrophages, granulocyte–macrophage colony-stimulating factor (GM-CSF), and macrophage colony-stimulating factor (M-CSF); 2) preeclampsia-related pro-inflammatory cytokines, IL1β and TNFα, regulate the expression of GM-CSF and M-CSF in first trimester decidual cells (FTDCs) through the NF-κB pathway; 3) FTDCs-secreted GM-CSF and M-CSF polarize macrophages toward the M1 and M2 subtypes, respectively, with sustained enhancement of the phagocytic ability of M2 macrophages potentially related to the downregulation of “don’t eat me” receptor SIRPα [108]. Human placental mesenchymal stem cells can transform macrophages from M1 type to M2 type and exert immunosuppressive effects, partially mediated by soluble molecules acting on glucocorticoid and progesterone receptors [118].

Overall, in preeclampsia, trophoblast apoptosis is heightened, and its clearance by macrophages is weakened, either absolutely or relatively, necessitating the assistance of non-professional phagocytes. HEECs, as one of the cell types in close contact with trophoblasts, exhibit enormous phagocytic potential. Peng et al. confirmed that HEECs can engulf apoptotic trophoblast cells and secret pro-inflammatory IL6 and MCP1, which may accelerate preeclampsia occurrence and progression [94]. As mentioned in “Efferocytosis and endothelial cells” section, trophoblast debris falling from the placenta into maternal blood circulation is primarily removed by maternal pulmonary vascular endothelial cells. Interestingly, the phagocytosis of apoptotic trophoblast debris does not activate pulmonary vascular endothelial cells, whereas phagocytosis of necrotic trophoblast debris does [95]. This finding is inconsistent with Peng's conclusion, which we attribute to two possible reasons: first, the origin of endothelial cells involved in phagocytosis may differ; second, the method of inducing trophoblast apoptosis may vary, resulting in differences in the size and characteristics of apoptotic debris and leading to different reactions upon engulfment. In preeclampsia, trophoblasts mainly show necrotic characteristics. Their phagocytosis induces endothelial cell activation, monocyte adhesion, and increased secretion of pro-inflammatory factors IL6 and TGFβ. These circulating IL6 and TGFβ further activate endothelial cells throughout the body, exacerbating endothelial damage and inflammation in preeclampsia [96, 97]. The antioxidant vitamin C and the calcium channel blocker nifedipine can inhibit the activation of endothelial cells that phagocytose necrotic trophoblasts, reduce IL6 secretion, and provide a certain preventive or therapeutic effect on preeclampsia [98, 99].

### Efferocytosis and recurrent spontaneous abortion

Recurrent spontaneous abortion (RSA) is an immune-related reproductive disorder, defined as two or more consecutive spontaneous abortions before 20–24 weeks of gestation, with an incidence of about 2.5% [119]. Known pathogenic factors of RSA include chromosomal abnormalities, endocrine factors, and anatomical factors. However, approximately 50% of RSA cases have unknown causes, termed unexplained recurrent spontaneous abortion (URSA) [120]. Currently, URSA is believed to be related to immune dysregulation and inflammatory response, with an imbalance between M1 and M2 macrophages playing a significant role. In normal pregnancies, M2 macrophages are the dominant subtype. In contrast, in pregnancies with complications, the number of M1 macrophages increases, leading to the secretion of harmful pro-inflammatory factors [121]. Y. Liu et al. isolated and cultured macrophages from mice during early pregnancy and conducted RNA sequencing to look for differentially expressed genes in the M1 and M2 subtypes. They found that histone deacetylase 9 (Hdac9) was highly expressed in M2 macrophages. Knocking out Hdac9 promoted the transformation of macrophages into the M2 type, enhanced their phagocytic ability, and potentially reduced pregnancy loss rates in mice [122]. Bai et al. explored the regulatory effect of placental-derived exosomes on macrophages and discovered that miRNA-30d-5p from these exosomes polarizes macrophage to the M2 phenotype by targeting Hdac9. Moreover, the conditioned medium of macrophages treated with placental-derived exosomes induces trophoblast migration and invasion but inhibits endothelial cell tube formation and migration. These effects are crucial for maintaining maternal–fetal tolerance and spiral artery remodeling in early pregnancy [84]. The IL33/ST2 axis has been reported to regulate macrophage polarization in recurrent miscarriage. Y. Sheng demonstrated that IL33 secretion increases while ST2 expression decreases in RSA, leading to an M1 skew in dMφ but with enhanced phagocytic capacity. The possible reasons for the discrepancy between the phenotype and function of dMφ may be as follows: 1) When dMφ exhibit an M1 phenotype, levels of pro-inflammatory cytokines and ACs increase in the maternofetal immune microenvironment. To reduce the risk of embryo loss and minimize harm to the mother, dMφ improve their efferocytosis to prevent the release of self-antigens. If this compensatory mechanism is inadequate, adverse pregnancy outcomes inevitably occur. 2) Excessive efferocytosis might directly cause embryo loss, rather than through a secondary immune response, due to diminished IL33 levels [123]. Recently, the team found that IL33/ST2 imbalance-induced RSA may be associated with the metabolic reprogramming of macrophages. Efferocytosis-related metabolism of dMφ in RSA is biased towards OXPHOS, resulting in dysfunctional efferocytosis, aggravating the destruction of the IL33/ST2 signaling pathway, and ultimately forming a positive feedback loop that leads to pregnancy failure [124]. Obstetric antiphospholipid syndrome (OAPS) is an autoimmune disorder strongly associated with recurrent miscarriage and preeclampsia, defined by the presence of circulating antiphospholipid antibodies. A recent study indicates that disordered efferocytosis and metabolic reprogramming of macrophages play a significant role in the pathogenesis of this condition. Compared to healthy controls, the decidua of OAPS patients shows an increased proportion of macrophages and a reduced uNK cells. Furthermore, macrophages in OAPS exhibit heightened phagocytic activity, enhanced complement activation signaling, and a greater dependency on glycolysis [125].

## Conclusions and prospects

Efferocytosis, i.e., the phagocytosis of ACs, is a key event to ensure tissue repair and organ development. Efferocytosis consists of four steps: “find me”, “eat me”, “engulfment” and “digestion”, which closely cooperate to guarantee the silent clearance of ACs. The process of efferocytosis requires energy supplied by glycolysis or OXPHOS. These two metabolic pathways may be active at different stages of efferocytosis and their switching can influence the function and inflammatory status of phagocytes. Upon internalizing ACs, macrophages handle a substantial metabolite load that must be promptly excreted or degraded. This process can, in turn, regulate efferocytosis and immune response. In this review, we focus on the role of efferocytosis at the maternal–fetal interface and the immunometabolism during efferocytosis. However, many questions still need to be investigated. Firstly, studies of efferocytosis at the maternal–fetal interface are mostly limited to in vitro cellular experiments. It is necessary to conduct in vivo studies to explore the exact effects of efferocytosis in the specific placental environment, so as to provide ideas for the resolution of inflammation in efferocytosis-related pregnancy complications. Furthermore, the metabolic reprogramming of phagocytes in pregnancy complications, particularly in the context of efferocytosis, remains underexplored. Studies to date primarily focused on whether the metabolic profiles of macrophages in disease conditions favor glycolysis or oxidative phosphorylation and whether their efferocytosis capacity is enhanced or diminished. Nevertheless, the interplay between metabolic reprogramming and efferocytosis, along with the underlying regulatory mechanisms, is poorly understood. Similarly, the metabolic changes that occur in non-professional phagocytes (e.g., endothelial cells) during efferocytosis and their influence on pregnancy outcomes have not been studied. The relationship between macrophage catabolism of apoptotic cargo and pregnancy also represents an unexplored research area. Investigations could begin by examining enzymes or other factors involved in the catabolic process to determine their effects on efferocytosis, inflammation, and pregnancy outcomes. Finally, identifying the regulatory factors of efferocytosis at the maternal–fetal interface is critical for discovering potential biomarkers or therapeutic targets for obstetric disorders.

## Data Availability

No datasets were generated or analysed during the current study.

## Abbreviations

ABCA1: ATP-binding cassette transporter A1
AC: Apoptotic cell
AhR: Aryl hydrocarbon receptor
ARP2/3: Actin-related protein 2/3
BAI1: Brain angiogenesis inhibitor 1
Cal: Calreticulin
GAS6: Growth arrest-specific 6
CD14: Cluster of differentiation 14
CX3CR1: Fractalkine
DAN-PKcs: Catalytic subunit of DNA-dependent protein kinase
DBL: Rac1 GTP-exchange factor
dMφ: Decidual macrophage
DNMT3A: DNA-methyltransferase-3A
DOCK180: Dedicator of cytokinesis protein 1
DSC: Decidual stromal cell
DUSP4: Dual-specificity phosphatase 4
ELMO1: Engulfment and cell motility protein 1
ERK: Mitogen-activated protein kinase 1
FAs: fatty acids
G2A: G-protein–coupled receptor
GM-CSF: Granulocyte-macrophage colony-stimulating factor
GPR: G-protein coupled receptors
GULP1: GULP PTB domain containing engulfment adaptor 1
Hdac9: Histone deacetylase 9
HEEC: Human endometrial endothelial cell
HMEC: Human microvascular endothelial cell
ICAM3: Intercellular adhesion molecule 3
IL33: Interleukin 33
IDO1: Indoleamine 2,3 dioxygenase 1
LAMP1/2: Lysosome-associated membrane protein 1/2
12/15-LOX: 12/15-Lipoxygenase
LPC: Lysophosphatidylcholine
LRP1: Low-density lipoprotein receptor (LDLR)-related protein 1
LXR: Liver X receptor
MCP1: Monocyte chemoattractant protein 1
M-CSF: Macrophage colony-stimulating factor
MFGE8: Milk fat globule epidermal growth factor-factor 8
mTORC2: CREB-regulated transcription coactivator
ORP1: Oxysterol-binding protein–related protein 1
PBX1: Pre-B cell leukemia transcription factor 1
PE: Preeclampsia
PFKFB2: 6-phosphofructo-2-kinase/fructose-2,6-bisphosphatase 2
PGE2: Prostaglandin E2
PI3P: Phosphatidylinositol 3-phosphate
PPARγ: Peroxisome proliferator activated receptor γ
PtdSer: Phosphatidylserine phosphate
PTGS2: Prostaglandin-endoperoxide synthase 2
P2Y2: Nucleotide receptors
RAGE: Receptor for advanced glycation end products
RILP: Rab7-interacting lysosomal protein
RSA: Recurrent spontaneous abortion
SAM: S-adenosylmethionine
sHLAG5: Soluble human leukocyte antigen G 5
SLC: Solute carrier family
SPMs: Specialized pro-resolving mediators
S1P: Sphingosine-1
S1PRs: Sphingosine-1 phosphate receptors
SIRT1: Sirtuin1
ST2: IL1RL1 receptor
TAM: Family of receptor tyrosine kinases (including TYRO3, AXL, and MerTK)
TIM1/3/4: T cell immunoglobulin and mucin domain1/3/4
V-ATPase: Vacuolar ATPase
VIP: Vasoactive intestinal peptide
WAVE1: Verprolin homology domain-containing protein 1
